# Impact of deforestation and climate on the Amazon Basin’s above-ground biomass during 1993–2012

**DOI:** 10.1038/s41598-017-15788-6

**Published:** 2017-11-15

**Authors:** Jean-François Exbrayat, Yi Y. Liu, Mathew Williams

**Affiliations:** 1 School of GeoSciences and National Centre for Earth Observation, University of Edinburgh, Edinburgh, UK; 2 School of Geography and Remote Sensing, Nanjing University of Information Science and Technology, Nanjing, China

## Abstract

Since the 1960s, large-scale deforestation in the Amazon Basin has contributed to rising global CO_2_ concentrations and to climate change. Recent advances in satellite observations enable estimates of gross losses of above-ground biomass (AGB) stocks due to deforestation. However, because of simultaneous regrowth, the net contribution of deforestation emissions to rising atmospheric CO_2_ concentrations is poorly quantified. Climate change may also reduce the potential for forest regeneration in previously disturbed regions. Here, we address these points of uncertainty with a machine-learning approach that combines satellite observations of AGB with climate data across the Amazon Basin to reconstruct annual maps of potential AGB during 1993–2012, the above-ground C storage potential of the undisturbed landscape. We derive a 2.2 Pg C loss of AGB over the study period, and, for the regions where these losses occur, we estimate a 0.7 Pg C reduction in potential AGB. Thus, climate change has led to a decline of ~1/3 in the capacity of these disturbed forests to recover and recapture the C lost in disturbances during 1993–2012. Our approach further shows that annual variations in land use change mask the natural relationship between the El Niño/Southern Oscillation and AGB stocks in disturbed regions.

## Introduction

The terrestrial carbon sink helps offset about 25% of anthropogenic emissions of fossil-fuel responsible for climate change^1,2^. While tropical forests are a major contributor to this sink, recent large-scale deforestation has weakened the capacity of the Amazonian forest to remain a long-term carbon store. The extent of land cover change in the Amazon Basin can now be quantified with some degrees of confidence using satellite-based observations^3^. Merging these observations with maps^4,5^ of Aboveground Biomass Carbon (AGB) provides a baseline estimation of gross losses from deforestation^6^. However, corresponding emissions may be partially compensated by regrowth in previously cleared areas^1^ while climate change, and extremes in particular, may alter the capacity of Amazonian forests to sequester C^7^. Therefore, estimates of the long-term net impact of large-scale deforestation and degradation on the land carbon sink, and its potential for recovery, are challenging to establish.

A way to address these problems is to study the deviation of current AGB stocks from potential stocks, to determine and separate the human-induced and climate-induced biomass deficits. These potential stocks are those that would exist under current climate if previous large-scale deforestation and degradation had not occurred (potential AGB further noted as AGB_pot_^8^; see Methods). AGB_pot_ can also be considered as a measure of local suitability for long-term carbon storage to inform reforestation and afforestation mitigation strategies. While it is not a directly measurable quantity, AGB_pot_ is comparable to carbon stocks predicted by terrestrial ecosystem models that omit land use and land cover change activities^8^ (such as those participating in the Intersectoral Impact Model Intercomparison Project, ISI-MIP^9–11^).

In a previous study^8^, maps of AGB_pot_ have been reconstructed over the Amazon Basin based on the relationship between climate^12^ and maps of observed AGB in the tropics^4,5^ (AGB_obs_) inside Intact Forest Landscapes^13^ (IFL). This study estimated a current human-driven AGB deficit (AGB_def_ = AGB_pot_ − AGB_obs_) ranging from 7.3 to 8 Pg C, or 11.6–12.2% of the basin-wide AGB_pot_. However, this previous approach relied on AGB_obs_ derived from data amalgamated over several years, which prevented any analysis of the evolution of AGB_def_. Indeed, AGB_def_ continuously evolves through time as it is the difference between AGB_pot_, which is only driven by climate and atmospheric CO_2_ concentrations, and AGB_obs_ which is driven by land use activities as well as climate and atmospheric CO_2_ concentrations. For example, anthropogenic activities such as deforestation (reforestation) may lead to a decrease (increase) in AGB_obs_ stocks, resulting in positive (negative) trend in AGB_def_. Meanwhile, the CO_2_-fertilization effect may lead to a greater potential for forest regeneration (i.e. greater AGB_pot_) as recent findings indicate it is the main driver of a global greening of the land surface^14^. However, locally changing climate conditions may lead to a reduction of the resilience of tropical forests and a transition toward less densely vegetated savannah landscapes^15^. There is a projected risk of Amazon die-back^7^ due to climate change, albeit with large uncertainty on its occurrence and severity^16^. It would reduce the potential for biomass recovery associated with reforestation by the end of the 21^st^ century. Therefore, it is important to estimate the resilience of AGB_pot_ to climate change to design efficient climate mitigation strategies based on reforestation.

In this study, we build on a previous approach^8^ (see Methods) to address the evolution of AGB_pot_, and hence AGB_def_, using a new dataset^17^ that provides annual estimates of AGB_obs_ from 1993 to 2012 at a 0.25° spatial resolution. By doing so, we aim to answer the following questions:

- How did AGB_def_ evolve in disturbed regions of the Amazon Basin over these two decades?
- Can we apportion this evolution to climate conditions affecting AGB_pot_ versus human activities reducing AGB_obs_?
- Would reforestation-based mitigation strategies be resilient to climate change in previously cleared regions of the Amazon Basin?

## Results

**Figure 1:**
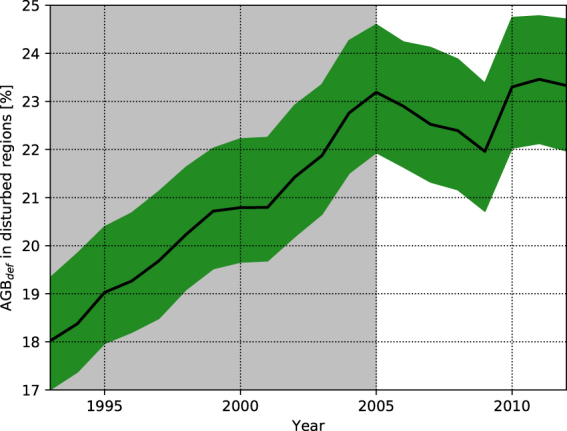
Time series of AGB_def_ in disturbed areas of the Amazon Basin expressed as a fraction of AGB_pot_. The green area represents the 5^th^ and 95^th^ percentile while the thick black line represents the mean. The shaded time period 1993–2005 highlights when the basin-wide increase in AGB_def_ exhibits a linear trend (r = 0.99; p ≪ 0.001) before this trend disappears after 2005.

**Figure 2:**
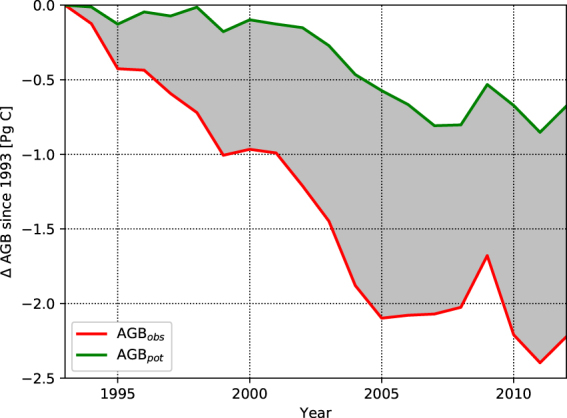
Change in total AGB_obs_ and AGB_pot_ in previously disturbed regions since 1993. Differences between AGB_pot_ and AGB_obs_, represented as a grey shading, correspond to the evolution of AGB_def_ for 1993–2012. For clarity only the mean estimates are represented.

We estimate a change in AGB_obs_ from 26.3 Pg C (with a 4.1 Pg C confidence range) in 1993 to 24.1 Pg C (with a 3.9 Pg C confidence range) in 2012, or a 2.2 Pg C (with a 0.2 Pg C confidence range) loss in regions of the Amazon basin which are not IFL. Using the machine-learning approach we derive a reduction of AGB_pot_ from 32.1 Pg C (with a 4.0 Pg C confidence range) in 1993 to 31.4 (with a 3.9 Pg C confidence range) in 2012 in the same regions. Comparing the evolution of AGB_obs_ and AGB_pot_ results in a human-driven increase in AGB_def_ from 18.0% (AGB_def_/AGB_pot_) in 1993 (with a 2.3% confidence range) to 23.3% in 2012 (with a 2.7% confidence range). Overall, ~1.5 Pg C of the ~7.3 Pg C mean AGB_def_ in 2012 was generated by combined anthropogenic activities and climate patterns since 1993 (Table 1). The evolution of AGB_def_ is strongly linear during 1993–2005 (*r* = 0.99; p ≪ 0.001) before plateauing from 2005 onwards with no significant trend (Fig. 1). The stabilisation of AGB_def_ after 2005 is associated to a reduction of AGB_obs_ loss rate from 0.17 Pg C y^−1^ (with a 6% relative uncertainty) to 0.04 Pg C y^−1^ (with a 14% relative uncertainty) before and after 2005 respectively (Fig. 2). It corresponds to a reduction in deforestation rates over the Brazilian Amazon seen in data from INPE (Figure S1 in the Supplementary Information; r = 0.97; p ≪ 0.001) while the smooth decreases of AGB_pot_ throughout the study period indicates a long-term negative impact of climate on the regeneration potential of disturbed regions (Fig. 2).

**Table 1:** Total AGB_obs_ in the disturbed regions of the Amazon Basin from Liu *et al*. (2015) and AGB_pot_ from this study in 1993 and 2012. Reported values are mean, with 5^th^ and 95^th^ percentiles between brackets. All values are in Pg C, rounded to the first decimal.

1993			2012		
AGB_obs_	AGB_pot_	AGB_def_/AGB_pot_	AGB_obs_	AGB_pot_	AGB_def_/AGB_pot_
26.3 (24.0/28.1)	32.1 (29.8/33.8)	18.0% (17.0%/19.3%)	24.1 (22.0/25.9)	31.4 (29.2/33.1)	23.3% (22.0%/24.7%)

**Figure 3:**
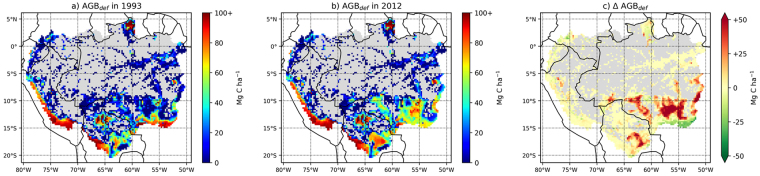
Aboveground Biomass Carbon deficit (AGB_def_) in (**a**) 1993, (**b**) 2012 and (**c**) the change in AGB_def_ over these two decades (**c**). Untouched IFL areas are represented in grey. In sub-panel c, positive (red) values indicate an erosion of AGB stocks while negative (green) values indicate a partial recovery. Maps were created using the cartopy module version 0.12.0 (http://scitools.org.uk/cartopy/) for python 2.7 (http://www.python.org/).

**Figure 4:**
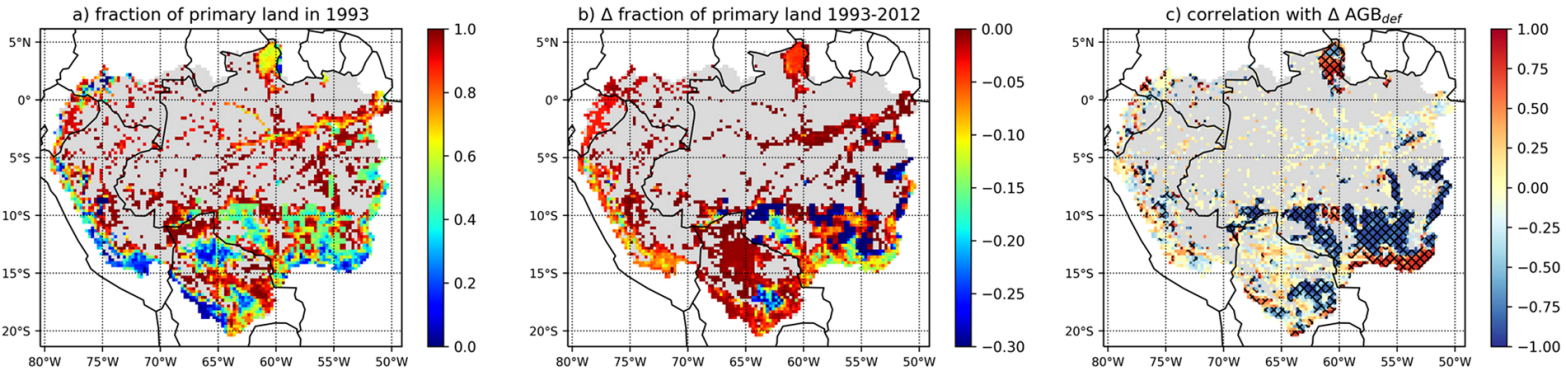
(**a**) Fraction of primary land outside IFL regions in 1993. Grey areas represent IFL regions. (**b**) Change in fraction of primary land between 1993 and 2012. Blue represents the decline in primary land during 1993–2012. (**c**) Temporal correlation between fraction of primary land and AGB_def_ from 1993 through 2012 over each 0.25° grid cell. Hatched areas represent statistically significant correlation (p < 0.05). A negative correlation indicates an increase in AGB_def_ (i.e. an erosion of AGB stocks) when the fraction of primary land decreases through time. Maps were created using the cartopy module version 0.12.0 (http://scitools.org.uk/cartopy/) for python 2.7 (http://www.python.org/).

The increase in AGB_def_ is heterogeneously distributed across disturbed areas of the basin (Fig. 3). While the spatial distributions of AGB_def_ are significantly correlated (*r* = 0.89; p ≪ 0.001) in 1993 (Fig. 3a) and 2012 (Fig. 3b), AGB_def_ increased by more than 50 Mg C ha^−1^ in some parts of the Brazilian arc of deforestation (between 10°S and 15°S; Fig. 3c) and in central Bolivia (south of 15°S; Fig. 3c). We note a reduction in AGB_def_, i.e. a recovery of AGB_obs_ stocks toward AGB_pot_, in the south-eastern edge of the basin, and to a lesser extent in northern Brazil. This recovery indicates that non-primary vegetation, mostly rangeland in these regions, may have built up biomass stocks from 1993 to 2012. Over the period 1993–2012, local increases in AGB_def_ can be explained by the erosion of primary land (Fig. 4). Conversely, local recovery of stocks associated to decreases in AGB_def_ corresponds to regions where the fraction of primary land was already low in 1993. This pattern indicates a recovery of AGB stocks in other land cover types, principally rangelands (Figure S2). Despite this apparent recovery of AGB stocks, the deficits in these regions were still > 50 Mg C ha^−1^ in 2012.

Our estimates indicate a significant negative correlation between inter-annual variations of the El Niño/Southern Oscillation (ENSO), represented by a winter composite of the Multivariate ENSO Index (MEI_w_, see methods) and detrended ΔAGB_pot_ integrated over previously disturbed regions (Figure S3 in the Supplementary Information; r = −0.57; p ≈ 0.01). This relationship indicates that negative (La Niña) phases of ENSO would drive positive anomalies in ΔAGB_pot_, i.e. a stronger sink, while positive (El Niño) phases of ENSO are associated with negative anomalies in ΔAGB_pot_, a weaker sink. However, past and current human activities mean that this significant relationship between ENSO and the sink strength disappears when comparing with de-trended ΔAGB_obs_ (r = −0.38, p > 0.10). We conclude that, through clearing and subsequent regrowth, human activities have become the main driver of inter-annual variability of the land-based sink, dominating natural climate drivers, in disturbed regions of the Amazon.

## Discussion

The annual biomass maps have allowed resolution of AGB changes across the Amazon Basin, indicating areas of heavy losses, but also some areas of AGB gain (Fig. 3). By mapping the potential biomass, we show the evolution of the basin’s capacity to store C, a baseline without human impacts. Because AGB_pot_ is determined from annual AGB_obs_ data in IFL, the annual variation in AGB_pot_ indicates the effect of climate on the storage capacity of the intact forest. We show that this potential has declined over 1993–2012 (Fig. 2) similarly to AGB stocks in IFL (Figure S4 in the Supplementary Information), due to climate and in spite of rising atmospheric CO_2_ concentrations (Table 1). Indeed, the evolution of AGB stocks in IFL is significantly correlated with the vegetation water stress estimated by GLEAM^18^ (r = 0.64; p < 0.01). The post-2005 decrease in AGB stocks in IFL follows a transition to stronger stress conditions around 2002 that prevail until the end of the study period in 2012. This transition toward more water-stressed conditions corresponds to the onset of the 2002–2003 El Niño episode^19^ followed by the 2005 and the 2010 Amazonian droughts^20,21^. Overall, these results indicate that drying conditions have degraded the capacity of the disturbed regions to regain their lost biomass which is line with the projected risk of climate driven Amazon biomass loss^7^. This climate-driven reduction in the capacity for regeneration also corroborates with risks for tropical forests to be replaced by savannahs if drier conditions dominates^15^.

Our results are first-order estimates and we are aware that hard-to-quantify and potentially large uncertainties may arise from ground-level measurements^22^, the way they are used in combination with remote-sensing data to derive large-scale biomass maps^23^, and the identification of forest cover^24^ and intact forest landscapes^13^. Therefore, we have validated the robustness of our machine-learning approach in several ways. First, it simulates annual AGB_obs_ with <0.1% bias integrated over out-of-sample IFL regions (Figure S5a in the Supplementary Information). We note a tendency to overestimate AGB in less densely vegetated regions (Figure S5b,c in the Supplementary Information) but the local mean relative bias is <1.2%. Second, pixel to country-scale estimates of the evolution of AGB_def_ through time are in agreement with independent datasets of deforestation (Figure S1) and land cover change rates (Fig. 3). Finally, the ~7.3 Pg C AGB_def_ estimated after 2005 is similar to the one reported previously^8^. Our highest confidence results indicate a ~0.08 Pg C y^−1^ increase in AGB_def_ for the period 1993–2012. This net number is about half of recent estimates of gross C emissions from the Amazonian deforestation^25^. It is in agreement with the ~50% compensation of gross C emissions from tropical deforestation by regrowth^1^. Assuming that large-scale deforestation started in 1960 (ref.^26^), the initial AGB_def_ of ~5.8 Pg C in 1993 corresponds to a higher 0.18 Pg C y^−1^ net biomass loss prior to this date. The decrease in AGB_def_ growth rate between 1993 and 2012, and especially after 2005 (Fig. 1), matches reports of a slowing down of Brazilian deforestation during 2005–2012 (refs^26–28^) but is also a result of a decrease in AGB_pot_ in disturbed regions of the Amazon Basin.

Furthermore, field studies^20,21^ and airborne measurements^29^ have shown that climate variability, and especially El Niño-induced droughts, have a large impact on the carbon balance of undisturbed areas of the Amazon Basin. These previous results are in agreement with the negative correlation between MEI_w_ and ΔAGB_pot_ (Figure S3 in the Supplementary Information). Overall, human-induced clearing and recovery processes mask the natural response of ecosystems to climate in disturbed parts of the Amazon Basin. While this impact is intuitive, we are able to demonstrate it quantitatively with the AGB_pot_ reconstructions. Finally, this result raises concerns on the viability of climate change mitigation strategies, as climate change is likely to challenge the resilience of forested landscapes.

## Conclusion

We have recreated annual maps of potential AGB for the Amazon Basin, which allows the net impacts of global change on basin biomass to be determined. Compared to maps of historical biomass, these indicate an increase of ~1.5 Pg C in the biomass deficit (AGB_def_) for 1993–2012. This basin-wide number is a net estimate of climate-induced variation of AGB_pot_ and deforestation-induced erosion of AGB stocks, which are partly compensated by regrowth in some areas post-deforestation. Overall, our results indicate that land use change continues to erode the carbon storage of the Amazon basin while climate change is impairing its capacity to sequester carbon through natural processes of regrowth, raising concerns on the long-term resilience of land-based mitigation strategies.

## Methods

### Annual maps of AGB

We use annual Above Ground Biomass maps^17^ (AGB_obs_) for the period 1993 through 2012 based on the passive microwave observed vegetation optical depth (VOD, dimensionless) from a series of satellites. VOD is an indicator of the total water content in the aboveground vegetation, i.e. including both canopy and woody components^30–32^. This VOD dataset can qualitatively capture the long-term and inter-annual variations in vegetation water content over different land cover types^33–37^. Annual AGB_obs_ maps were created by establishing a relationship between VOD and a pan-tropical map^4^ of AGB_obs_ circa 2000. These annually resolved maps are comparable with previous independent estimates of AGB dynamics^1,5,6^. For more details about the methodology used to create AGB_obs_ maps, please refer to Liu *et al*. (2015, ref.^17^).

### Creating potential AGB maps

To derive the evolution of the AGB deficit (AGB_def_) we first created annually resolved maps of potential Above Ground Biomass (AGB_pot_) in previously disturbed regions. AGB_pot_ corresponds to AGB stocks there would exist under current climate if deforestation had not occurred in these regions. It can also be conceptualized as the current forest regeneration potential if regrowth was instantaneous. The method to create AGB_pot_ maps was described in Exbrayat and Williams (2015; ref.^8^) and is only briefly summarized hereafter.

First, we used a Random Forest machine-learning algorithm^38,39^ to reproduce AGB_obs_ as a function of climatology in identified Intact Forest Landscapes (IFL) which cover about 55% of the Amazon Basin. The Random Forest technique relies on multiple decision trees (here n = 1,000) to group data points as a function of driving data. Then, in each final node a multiple linear regression is trained to predict the target variable (here AGB_obs_) as a function of explanatory data. Each individual decision tree is trained on a randomly selected subset of the data and the final prediction is the average of all trees. Here, we use the CRU CL2.0 climatology dataset^12^, re-gridded to a matching 0.25° resolution with the Climate Data Operators version 1.6.9, and latitude, a proxy of intra-annual photoperiod amplitude, as explanatory variables to predict AGB in IFL. The assumption is made that regions identified as ‘intact’ may be subject to small-scale indigenous management^40^ or disturbances^41^ that are negligible at the coarser 0.25° resolution used here^8^. Compared to our previous study we used an updated IFL dataset^13^ that represents the extent of intact regions for the year 2013. It ensures that training regions have remained intact throughout the whole period covered by the AGB_obs_ dataset (i.e. 1993–2012). In addition to these continuous drivers, we used a categorical variable to separate pixels corresponding to large-scale open water regions in the Global Lakes and Wetlands Database^42^. As VOD values are strongly influenced by the open water dynamics, the pixels with large-scale open water are identified and the VOD values over these pixels are assumed constant among different years^17^.

Once trained the algorithm can then be used to estimate annual, climate-driven, AGB_pot_ in previously disturbed regions (i.e. outside IFL) regions. Although it has been identified as the major driver of the recent greening of the land surface^14^, CO_2_ is not explicitly used in our approach because of the lack of availability of spatially-explicit data of atmospheric concentrations. However, we assume that the impact of increasing CO_2_ on AGB stocks is intrinsically included in time series of AGB in IFL which also include the impact of changing climatic conditions. Using annual maps of AGB_pot_ we can calculate an AGB deficit (AGB_def_ = AGB_pot_ − AGB_obs_) and derive time series of its evolution from 1993 to 2012. As the temporal evolution of AGB_pot_ is only driven by climate and atmospheric CO_2_ concentrations, we assume that AGB_def_ is representative of the net and cumulative impact of anthropogenic activities on biomass dynamics on AGB stocks. We perform the analyses using the mean AGB_obs_ from Liu *et al*. (ref.^17^) to derive AGB_pot_ and AGB_def_. Furthermore, we evaluate the uncertainty in our approach by performing the analysis with the 5^th^ and 95^th^ percentiles of AGB_obs_ data^17^ to report the corresponding confidence ranges in AGB_pot_ and AGB_def_. As a proof of concept, we first validate the method using ~50% of randomly selected pixels in IFL as training dataset and the remaining IFL pixels as target dataset to assess the robustness of the approach to recreate 20 years of AGB_pot_. Corresponding results are presented in Figure S5 of the supplement. We note a good agreement between reconstructions and data in IFL although there is a tendency for the machine-learning to overestimate AGB in less densely vegetated regions.

### Validation of results

Our estimates of AGB_pot_ cannot be directly validated against field data. However, we expect the temporal evolution of AGB_def_ to be related to contemporary deforestation rates and land cover changes. Therefore, we compare time series of AGB_pot_ from pixel to country-scale with independent datasets of Land Use and Land Cover Change (LULCC). First, we compare annual deforestation rates reported by INPE for the Brazilian part of the Amazon Basin with the corresponding trend in AGB_def_ over the whole period 1993–2012. Second, we use spatially-explicit data from the Land-Use Harmonization project version 2 (LUH2v2h; data updated from ref.^43^). LUH2v2h is a global driving dataset that provides annual land cover information for the period 850–2015 C.E. in the Land Use Model Intercomparison Project^44^ (LUMIP) contribution to the upcoming sixth phase of the Coupled Model Intercomparison Project^45^ (CMIP6). In LUH2v2h land covers are distributed between 12 classes (2 primary land classes, 2 secondary land classes, 5 cropland classes, 2 pasture and rangeland classes and 1 urban class) and the fraction they cover in each 0.25° pixel is reported annually.

### Climate sensitivity

We compare the evolution of AGB_obs_ in IFL with time series of the vegetation stress factor S from the GLEAM dataset v 3.1a (ref.^18^). GLEAM is a data-assimilation system that uses satellite observations to constrain daily estimates of global terrestrial evaporation and root-zone soil moisture^46^. The factor S is an output of GLEAM and represents the ratio of actual evapotranspiration to potential evapotranspiration, an indicator of ecosystem’s water stress. It is as a function of vegetation state and soil moisture availability and therefore takes long-term effects of precipitation conditions into account. We use the mean annual value of S across the IFL regions of the Amazon Basin, expressed as a z-score, to explain the evolution of AGB_obs_ (Figure S4).

We seek to further understand the impact of large-scale human disturbances by quantifying their impact on the response of ecosystems to climate variability. We focus on the El Niño/Southern Oscillation (ENSO), a main driver of global climate variability^47^. The state of ENSO, quantified through the calculations of an index, significantly correlates with the strength of the global land carbon sink^48^. Indeed, positive (negative) El Niño (La Niña) phases drive warmer and drier (cooler and wetter) conditions over large parts of the pan-tropical region, including the Amazon Basin, which explains spatial patterns of ecosystem carbon uptake^48^. Following previous studies^48,49^ we use a winter composite of the Multivariate ENSO Index^50,51^ calculated between Dec/Jan and Mar/Apr (referred as MEI_w_). To quantify the impact of human disturbances on the response of the Amazon terrestrial carbon sink to ENSO, we study the correlation between MEI_w_ and detrended anomalies of annual ΔAGB_obs_ and ΔAGB_pot_ stocks integrated over disturbed (i.e. non-IFL) regions of the Amazon Basin. We choose to rely on a global index rather than actual data of temperature and precipitation for the Amazon Basin because past deforestation may have altered these quantities in regions where land-atmosphere coupling is strong^52,53^.

### Data availability

The data generated during this study are available from the corresponding author on reasonable request.

## Electronic supplementary material

Supplementary Figures
