# Supplementary material for: Impact of deforestation and climate on the Amazon Basin’s above-ground biomass during 1993–2012
Source: Sci Rep. 2017 Nov 15;7:15615. doi: 10.1038/s41598-017-15788-6 (PMC5688063; doi:10.1038/s41598-017-15788-6)
Supplement: Supplementary file 1 — Supplementary Figures [file 41598_2017_15788_MOESM1_ESM.pdf]

Impact of deforestation and climate on the Amazon Basin's above-ground biomass during 1993-2012

Supplementary Information

Jean-François Exbrayat[1]*, Yi Y. Liu[2,3] and Mathew Williams[1]

[1] School of GeoSciences and National Centre for Earth Observation, University of Edinburgh, Edinburgh UK

[2] School of Geography and Remote Sensing, Nanjing University of Information Science and Technology, Nanjing, China

[3] ARC Centre of Excellence for Climate System Science and Climate Change Research Centre, University of New South Wales, Sydney, NSW, Australia

*correspondence to: j.exbrayat@ed.ac.uk

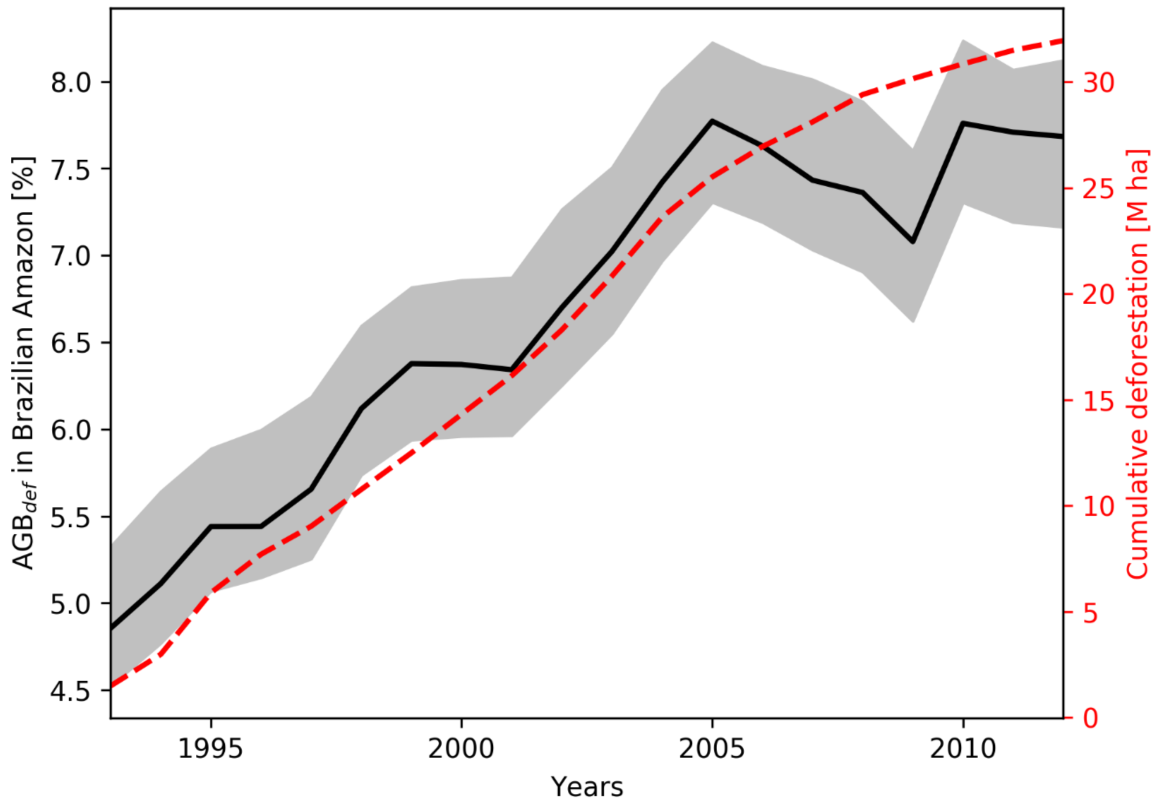

Figure S1. Evolution of the mean (black line) and 90% confidence range (grey area) of AGB$_{def}$ over the Brazilian Amazon compared to cumulative deforestation reported by INPE (red dashed line). Mean AGB$_{def}$ and deforestation rates strongly correlate (*r* = 0.97; p << 0.001).

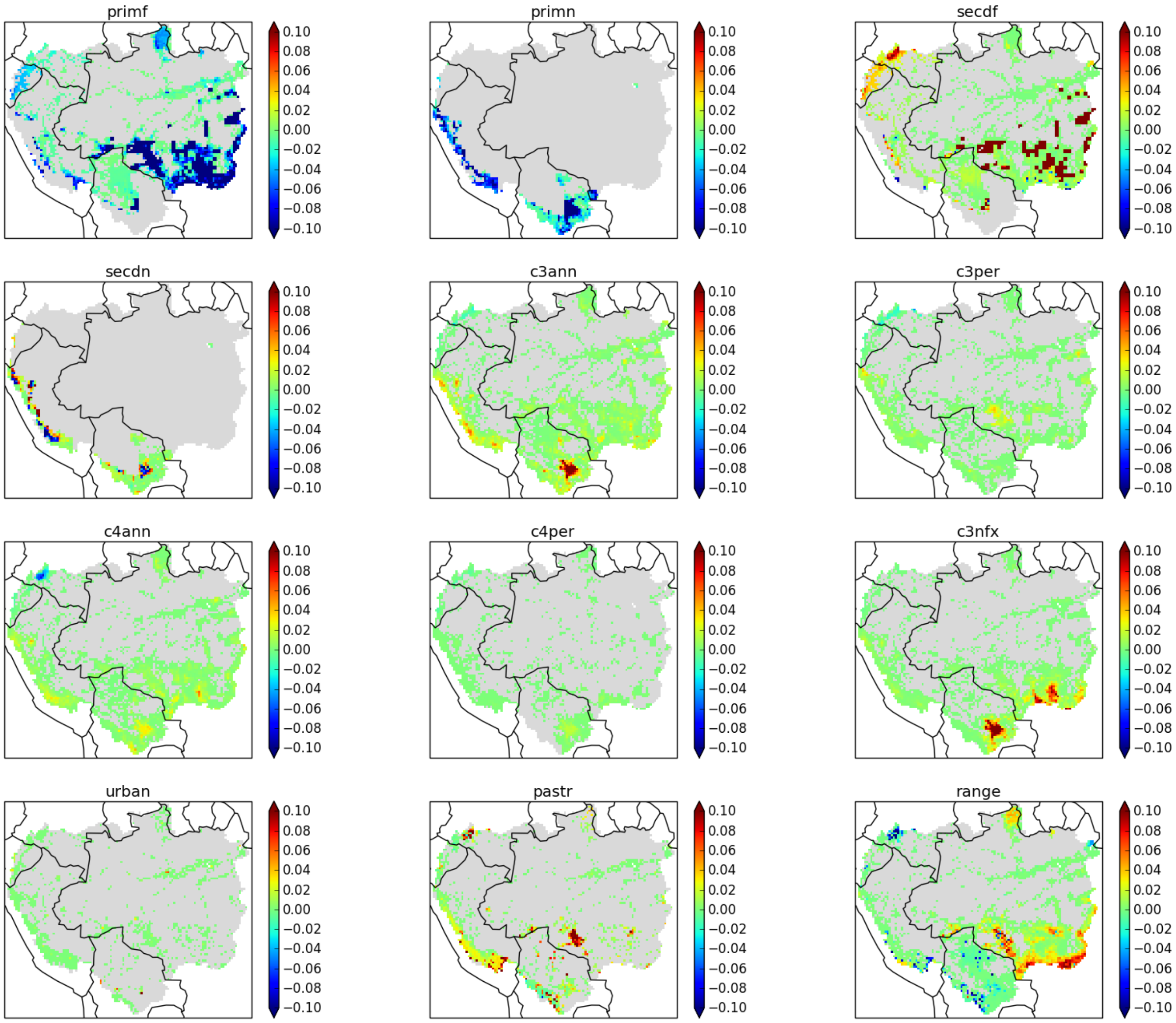

Figure S2. Change in the fraction covered by each land cover type in the LUHv2h database for the period 1993-2012. Maps were created using the cartopy module version 0.12.0 (http://scitools.org.uk/cartopy/) for python 2.7 (http://www.python.org/).

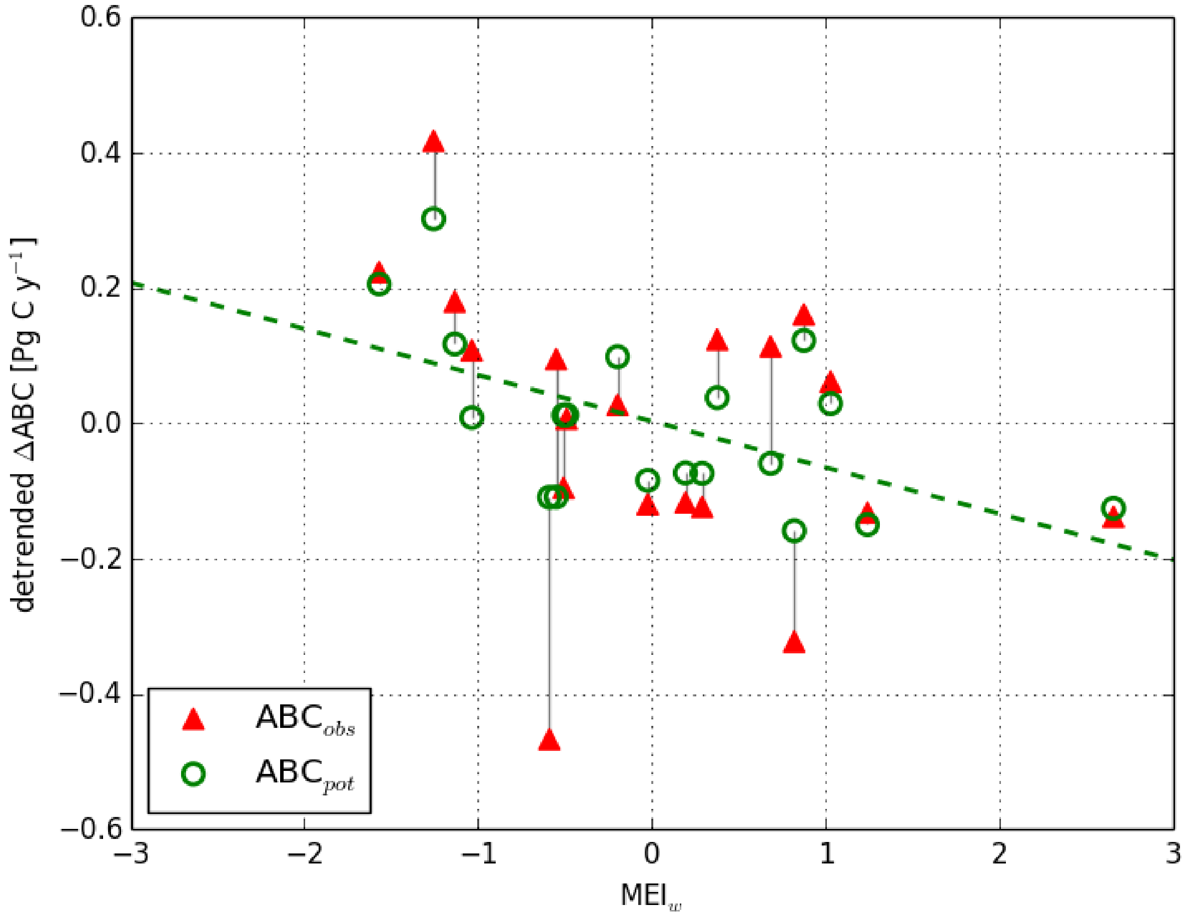

Figure S3. Relationship between MEIw and detrended inter-annual ΔABCobs and ΔABCpot in disturbed regions for 1993-2012. The dashed line represents the linear significant negative correlation between annual ΔABCpot and MEIw ($r = -0.57$; $p \approx 0.01$) while the non-significant relationship between ΔABCobs and MEIw ($r = -0.38$; $p > 0.10$) is not represented. Vertical lines link data points corresponding to the same years for better visualization.

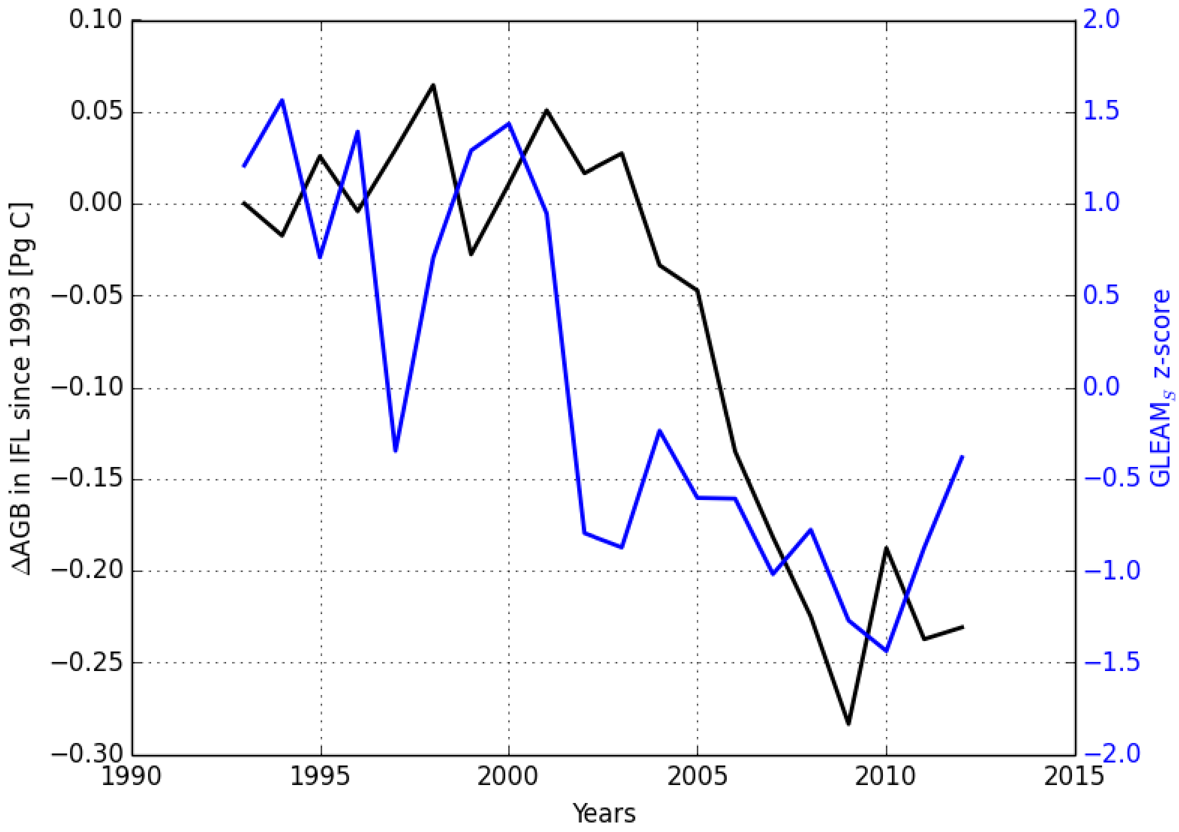

Figure S4. Evolution of AGB stocks in IFL regions of the Amazon Basin since 1993 in the dataset from Liu et al. (2015; in black) and mean annual value of the GLEAM$_S$ stress factor, as z-score, for the corresponding regions (blue). The stress factor S represents the ratio of actual evaporation to potential evaporation and is a function of vegetation state and soil moisture availability designed to take the effect of cumulative changes in precipitation patterns (Martens et al., 2017). Therefore, negative (positive) z-score equals stronger (higher) water stress on vegetation.

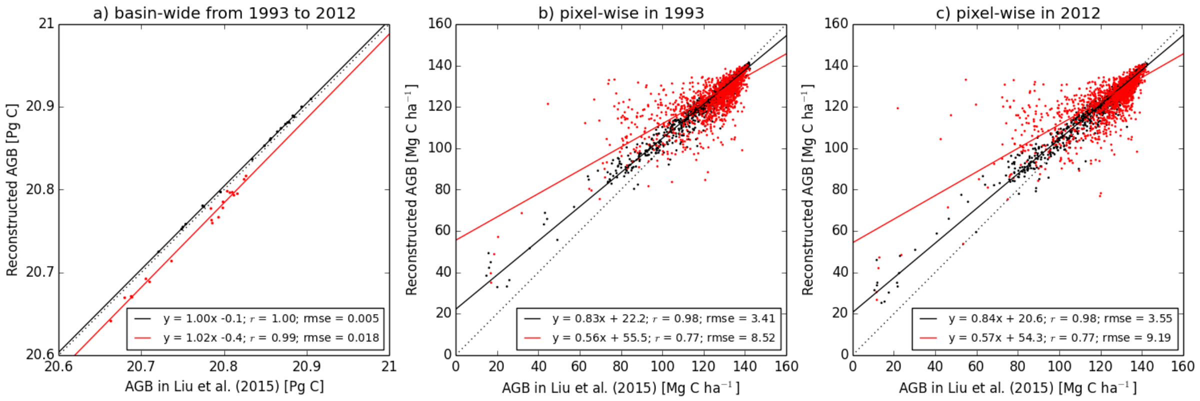

Figure S5. Validation of the Random Forest approach to reproduce AGB in IFL: basin-wide stocks (a), pixel-wise in 1993 (b) and 2012 (c). Training data consist of 2136 randomly selected pixels in the IFL of the Amazon Basin while validation data was extracted from 2127 out of sample validation points. All relationships are significant with p << 0.001 and yield absolute relative biases < 1.2%.
